# Triple negative breast cancer: the kiss of death

**DOI:** 10.18632/oncotarget.16938

**Published:** 2017-04-07

**Authors:** Adriana-Andreea Jitariu, Anca Maria Cîmpean, Domenico Ribatti, Marius Raica

**Affiliations:** ^1^ Department of Microscopic Morphology/Histology, Angiogenesis Research Center, Victor Babeş University of Medicine and Pharmacy, Timişoara, Romania; ^2^ Department of Basic Medical Sciences, Neurosciences and Sensory Organs, University of Bari Medical School, Bari, Italy; ^3^ National Cancer Institute “ Giovanni Paolo II”, Bari, Italy

**Keywords:** triple negative breast cancer, progression, metastases, therapeutic targets

## Abstract

One of the most controversial women malignancies, triple negative breast cancers (TNBCs) are critically overviewed here, being focused on data useful in clinical practice or to improve the therapy and patients survival. TNBCs “choose” young women and its “kiss” is, unfortunately deadly in most cases. Currently, few sparse data are available in literature concerning the origins of TNBC. Vasculogenic mimicry detected in TNBCs, seems to be determined by a population of CD133+ cells and may be stimulated by different pharmacological agents such sunitinib. Despite the fact that TNBCs do not usually metastasize through the lymphatic pathways, TNBCs may be characterized by lymphatic invasion and by an increased lymphatic microvascular density. If TNBCs treatment depends on the molecular profile of the tumor, the same statement may be postulated for TNBCs metastasis. Whether metastases have a similar phenotype as the primary tumor remains an enigma. Therefore, the question: ‘Could TNBC be subject to a standardized, unanimously accepted therapeutic strategy or is it strictly subclass-dependent?’ remains to be further investigated.

## THE EMERGING CONCEPT OF TRIPLE NEGATIVE BREAST CANCER

Breast cancers are commonly associated with a high incidence and a high mortality rate in the female population worldwide. However, at a microscopic and molecular level, breast cancer is not a homogeneous disease, thus being the focus of numerous ongoing studies. The molecular heterogeneity of the normal breast tissue has been previously documented and has outlined the different molecular profiles of epithelial and non-epithelial cells responsible for the existence of several molecular types of breast carcinomas, already characterized [1]. Starting from the histopathological classification up to the molecular classification, breast cancer has been constantly redefined in order to ensure a better management of the patient. In 2012 Boyle et al. stated that the minimal characterization of breast cancer was a “situation that had lasted for a century”, until “a quiet revolution has taken place so that in modern times breast cancer is characterized by its molecular and clinical heterogeneity” [2].

More than fifteen years ago, based on gene analysis, Perou et al. were the first to describe the molecular types of breast cancer [3]. Breast cancer was classified in relation to the molecular features of the mammary epithelium, namely: estrogen receptor positive (ER+)/luminal-like, basal-like, receptor tyrosine kinase positive (erb-B2+) and normal breast [3]. According to Perou et al. most of triple-negative breast cancers (TNBCs) were included in the basal-like subtype [2, 3]. Complementary to the previously mentioned study, Sorlie et. al have identified five major molecular types, that included the basal-like, the ErbB2-overexpressing, two luminal-like and a normal breast tissue-like subgroup [4, 5]. It has been stated that the different molecular types of breast cancer have distinct molecular mechanisms and act as biologically distinct entities that require a different therapeutic management [6]. In addition, the examination of BRCA1 carriers had lead to the statement according to which this particular genotype favors the occurrence of basal-like types [5]. Survival analysis revealed a poor prognosis in patients diagnosed with basal-like type while the two ER+ groups showed a variable outcome [4]. Whether TNBCs may or may not be completely included in the basal-like carcinoma remained a controversial hypothesis until, back in 2010, Foulkes et al. have pinpointed the fact that this molecular type “is often, but not always, a basal-like breast cancer” [7]. Moreover, a study conducted by Badve et al. revealed that the majority of TNBCs exhibit a basal-phenotype and most of the basal-like breast cancers show a triple-negative profile, although the two entities are not related [8]. TNBC has been characterized as exhibiting a negative profile for the three markers used in the molecular classification of breast cancer, ER-/progesterone receptor negative (PR-)/human epidermal growth factor receptor 2 negative (HER2-) [7, 9, 10]. Although more rarely diagnosed, this molecular type is known to be highly aggressive and seems to be mostly common for younger female population, and especially black women [8, 11].

## PATHOLOGICAL AND MOLECULAR CHARACTERIZATION OF TRIPLE NEGATIVE BREAST CANCER

As a distinct molecular entity, TNBCs appear to be quite heterogeneous at a histopathological level. They frequently show features of ductal invasive carcinomas [12] although metaplastic, medullary and apocrine features are also found [9, 13]. Moreover, TNBCs may present themselves as adenoid cystic lesions, histiocytoid carcinomas and even as invasive lobular carcinomas [13]. A relatively large number of breast cancers that do not exhibit a basal phenotype appeared to have a triple-negative profile [8]. Therefore, from a morphological and molecular point of view, TNBC may somehow be classified into four main categories that include the normal-like and the apocrine subtypes [8]. It appears that some histopathological features of breast cancer such as pleomorphic lobular carcinoma and mixed ductal-lobular carcinoma exhibit a triple-negative molecular profile [8]. Also, most invasive carcinomas that develop from microglandular adenosis areas are in fact triple-negative tumors [8, 13]. Recent studies have shown that metaplastic carcinomas are usually TNBCs [14]. Metaplastic carcinomas are known to be rare, aggressive diseases of the breast that are usually diagnosed at grade 3 and, similar to TNBCs, have no specific therapeutic guidelines [14, 15].

The histopathological features of TNBCs includes a high nuclear grade, increased mitotic activity, high nuclear-cytoplasmic ratio and accelerated tumor proliferation rate, although a low percentage of TNBCs have shown low grade features [9]. Both high grade and low grade TNBCs show different molecular features and are associated with specific chromosomal translocations that lead to complex gene fusion processes [13]. If the histopathological features of TNBCs are heterogeneous, their molecular presentation is even more controversial. The term “triple-negative breast cancer” seems somehow obsolescent, while “triple-negative breast cancers” appears to be more appropriate. Different types of TNBCs include the basal expression subtype, the p53 mutation associated to TNBC, the high-genomic instability subtype, TNBCs that associate phosphatidylinositol-3 kinase (PI3K) pathway activation and the non-basal or the p53 wild-type TNBC [16]. Also, TNBCs are sometimes associated with genetic mutations, others than p53 and BRCA1, such as alphaB-crystallin [17]. The molecular characteristics of TNBCs vary in case of both carriers and non-carriers of associated genetic mutations [18]. Patients with TNBCs that are associated with BRCA1 mutations show similar clinicopathological characteristics with patients diagnosed with other BRCA1-mutated tumors [19]. It appears that sporadic TNBCs, as well as basal phenotype tumors are closely linked to BRCA1 dysfunction which is highly implicated in the development and progression of this disease [20]. It has also been stated that the aggressive behavior of TNBCs may be linked to cellular senescence and cytoprotective autophagy that are able to enhance the malignant phenotype of this disease [21]. Dillon et al. have shown that TNBC is characterized by a wide range of genetic mutations such as myelocytomatosis oncogene (MYC) amplification and alterations of tumor protein p53 (TP53), Aurora kinase A (AURKA) and kinase insert domain receptor (KDR) [22]. Moreover, it appears that TNBCs that exhibit basal-like features are usually associated with MYC mutation [22]. In addition, dual specificity phosphatase 4 (DUSP4), with inhibitory effect on proliferation, migration, invasion and on cell growth in TNBCs, is either underexpressed or absent [23]. Genetic mutations that occur in TNBCs may be associated with different histopathological changes such as fibrosis and inflammation, as it has been demonstrated in TNBCs that overexpress toll-like receptor 9 (TLR9) [24].

An aspect that cannot be neglected in medical practice is that TNBCs are poorly differentiated tumors [25], thus making routine diagnosis a difficult task. Moreover, metastases deriving from TNBCs are hardly recognizable and are first considered metastases with unknown origin [25]. The establishment of a panel of markers with clinical usage for proper diagnosis of TNBCs metastases is quite the challenge. Davion et al. have shown that TNBCs usually exhibit a negative profile for most markers of breast origin [25]. However, the authors found a focally positive for cytokeratin 7 (CK7) which was heterogeneous [25]. According to several authors, prolactin induced protein (GCDFP-15) and mammaglobin (MAM) appear to be of limited utility in TNBCs, most of these tumors exhibiting a negative profile for either marker [25–27]. Transcription factors GATA binding protein 3 (GATA-3) seems to gain ground in the diagnosis of primary TNBCs and their metastases according to Huo et al., although further testing of this marker is required [26].

For a long period of time, TNBCs and basal-like breast carcinomas (BLBCs) have been regarded as overlapping molecular entities, associated with poor prognosis and aggressive behavior [28–30]. BLBCs show a positive expression for cytokeratin 5/6 (CK5/6) or epidermal growth factor receptor 1 (EGFR1) [28, 31], cytokeratin 14/17, caveolin 1 and 2, cyclin-D1, P-cadherin and are characterized by mutations in the p53 gene [29]. The discrimination between BLBCs and other molecular types of cancer included in the TNBC group is based on the gene expression profile of basal-like carcinomas which is similar to the basal-myoepithelial layer of the normal breast cells [29]. This immunohistochemical profile is supported by a study conducted by Li et al. where breast cancer specimens were classified into basal-like and normal-like groups depending on their expression for CK5/6 and EGFR [32]. Also, Wiese et al. have classified feline breast cancer specimens depending on the expressions of ER, PR, HER2, CK5/6 and EGFR into TNBCs and BLBCs [33]. BLBCs stand out as distinct molecular entities and are distinguishable from the molecular apocrine type (androgen receptor (AR)-positive and/or GGT-1-positive group), claudin low type (claudin 3-, claudin 4-, or claudin 7- negative and/or E-cadherin-negative group), mixed type (molecular features of more than two types), and the null type (showing none of the above mentioned features) [34]. Maeda et al. have shown that TNBC types that have a CK5/6(−)/AR(−)/p53(+) profile are of worst prognosis [31]. It appears that CK5/6 along with tumor proliferation index (Ki-67), lymph node status and tumor size is an independent prognostic factor in TNBCs [35]. Basal-like status is considered an important parameter of pathologic complete response (pCR) to neoadjuvant chemotherapy in locally advanced TNBCs [36]. As it has been stated by Alluri et al., BLBC ‘is an inherently and biologically more aggressive pattern of disease’ [37]. Prat et al. even state that breast cancer approach should consider “stratifying patients based upon basal-like (BL) *versus* non-basal-like (non-BL) gene expression profiles, which appears to be the main biological difference seen in patients with TNBC” [38]. Whether BLBCs should or should not represent the main parameter for patients stratification in TNBCs, remains to be further studied. However, it is certain that this molecular type is significantly associated with markers of poor prognosis such as p53 and hypoxia-associated factor (CA9) [39] and it also shows several abnormalities of the BRCA gene [40].

## IS TRIPLE-NEGATIVE BREAST CANCER DIAGNOSIS A DEATH SENTENCE?

TNBCs are usually diagnosed in the younger female population, under the age of 50, with an incidence that ranges between 10 % and 17 % or between 10 % and 24 % according to different statistics [8, 9]. 10% of the diagnosed TNBCs are characterized as grade I, although some scientific papers state that this disease has no grade I presentation [8]. As an aggressive molecular type, TNBCs present a high tendency to metastasize and patients are at a higher risk to relapse compared to other molecular types [9]. It appears that the most common sites of recurrence for TNBC are the lungs and the brain [41]. Along with its molecular heterogeneity, the greatest obstacle in the treatment of this disease is represented by the fact that it lacks a therapeutic target due to negative profile for PR, ER and HER2 [17, 21]. Patient prognosis, survival and response to therapy depend on the clinical and pathological presentation of this disease [13] and it is known to be poor overall. The spreading pathways of the malignant cells are quite variable, therefore no relationship has been found between TNBC and lymph node status, only in a low percentage of cases [13].

At the time being, no approved targeted therapy for TNBC is available in clinical practice [42, 43] although a great number of drugs are subject to research studies and clinical trials. The standard therapy for this molecular type of cancer includes agents such as taxanes, anthracyclines and cyclophosphamide [42] and is similar to other HER2- breast cancers [43]. Other therapeutic strategies include the use of poly ADP ribose polymerase inhibitors (PARP inhibitors), EGFR inhibitors, Src family kinase inhibitors and antiandrogens [43]. In a low percentage of triple-negative breast cancers, PI3K/AKT/mTOR pathway is activated, which lead to the hypothesis that inhibition of the PI3K signaling pathway may ensure a proper treatment for patients diagnosed with mesenchymal and luminal androgen receptor (LAR) types of TNBCs [43]. Also, platinum salts seem to be an emerging therapeutic option in TNBCs [43, 44]. Experimental studies have shown the benefits of platinum salts based therapy especially in patients who present BRCA1/BRCA2 mutations and in those with different genomic instabilities of TNBCs [43, 44]. Despite its aggressive behavior, TNBC seems to be amongst the few molecular types of breast cancers that are associated with a high density of the lymphocyte population [45]. It appears that tumor-infiltrating lymphocytes possess the ability to enhance the patients’ response the chemotherapeutic agents [45].

TNBCs are frequently diagnosed when reaching grade III, are large size tumors and are usually associated with specific metastatic patterns [46]. Several studies have pinpointed a large range of risk factors for TNBCs, besides the African-American origin and young age of the patient. These risk factors overlap those of breast cancer in general, namely, young age at menarche, young age at time of first birth, high parity, lack or lower duration of breastfeeding and abdominal obesity [46]. Also, patients diagnosed with ovarian cancer and/or patients who previously underwent radiotherapy and endocrine therapy have an increased risk of developing TNBCs [47]. However, unlike in cases of non-TNBCs cancers, female patients diagnosed with TNBC have a higher tendency of developing distant recurrences in about three years after being diagnosed and die in about five years following diagnosis [48]. Surprisingly, the risk of distant recurrences and death seems to decline and is maintained at a constant level after the mentioned periods of time [46]. Besides the large dimensions of the tumor, the high nuclear and histological grade, TNBCs present significant venous and lymphatic invasion, parameters that are associated with a poor long term outcome [49, 50]. Moreover, this molecular type of breast cancer is characterized by a higher proliferation rate, frequent visceral metastases and is associated with the worst outcome in patients that present lymph node metastases [51]. It appears that, along with HER2 + tumors, TNBCs show higher rates of recurrences in the central nervous system [52, 53]. TNBC may frequently determine metastases in the lungs, but the bones are rarely involved [53].

## QUESTIONABLE ISSUES IN TNBCS

Currently, only a few sparse data are available in literature concerning the origins of TNBC. However, it is certain that genetic instability along with different environmental factors promote TNBCs development. The researches that were focused on determining the mechanisms of carcinogenesis in TNBCs reveal quite controversial results that may be subject to further debate and scientific analysis. The study conducted by Caliari et al. on feline mammary lesions demonstrated a positive expression for vimentin, cytokeratin 14 (CK14) and CK5/6 in breast tumors that are negative for HER2 [54]. These particular tumor types may resemble the basal-like/claudin-low types of TNBCs diagnosed in human female patients [54]. TNBCs may present a potential bilineage progenitor through the diffuse CKs/vimentin co-expression observed in luminal cells of the non-neoplastic ducts [54]. Also, Caliari et al. have identified luminal and myoepithelial progenitor cells located in the duct system, cells that may represent the key players in TNBCs development [54]. Another hypothesis, that suggests the implications of miR-200 and miR-221/222 microRNA families in breast carcinogenesis, refers to the development of poorly differentiated tumor types in case of miR-221/222 overexpression [55]. It appears that TNBCs development is not limited to singular gene mutations or to disorders that concern one single cell population. The origins of TNBCs depend on its various molecular types and imply a great range of molecular mechanisms even for the same subtype. For example, TNBCs that present themselves as adenoid cystic carcinomas (AdCC), are genetically similar to AdCC of salivary glands, and involve several genes somatic mutations, including genes that encode fibroblast growth factor receptor 2 (FGFR2) [56]. Moreover, breast AdCC are associated with mutations regarding chromatin remodeling, cell adhesion and genetic alterations of different signaling pathways [56]. Amongst the signaling pathways that are implicated in TNBCs carcinogenesis, mammalian target of rapamycin (mTOR) with its two branches, mTORC1 and mTORC2, appears to be the most potent [57]. mTORC1 seems to possess more important implications as the major regulator of cell proliferation [57]. In some TNBCs types, such as the immune-activated subset, the JAK/STAT signaling pathway appears to exert control on cell proliferation [58].

Similar to other malignant tumors, hypoxia represents one of the major factors that determines the generation of cancer stem cells in TNBCs [59, 60]. As the unique ability of highly aggressive tumors to mimic the patterns of embryonic vasculogenesis, vasculogenic mimicry is present in triple-negative breast cancers [61, 62]. Vasculogenic mimicry detected in TNBCs, appears to be determined by a population of CD133+ cells and may be stimulated by different pharmacological agents such as sunitinib [59]. TNBCs are able to form vascular-like networks *in vivo* and *in vitro*, in xenograft models and in human specimens [62]. This phenomenon is strongly influenced by platelet-derived growth factor receptor beta (PDGFRbeta) and FGFR2 [62]. Vasculogenic mimicry determines the formation of blood lacunae surrounded by tumor cells, and represents and indicator of poor prognosis in TNBCs patients [62]. Besides vasculogenic mimicry, high levels of disintegrin and metalloproteinase 8 (ADAM8) are also associated with a poorer prognosis, due to its stimulating effects on tumor angiogenesis *via* vascular endothelial growth factor-A (VEGF-A), and on transendothelial cell migration *via* beta1-integrin activation [63]. VEGF-A influences TNBCs angiogenesis, thorough the STAT1/HIF-1alpha/VEGF-A signaling axis [64, 65]. VEGF-A overexpression in TNBCs patients is associated with a poor outcome [66]. Along with VEGF, angiopoetin-1 (Ang-1) and angiopoetin-2 (Ang-2) appear to be potent promoters of tumor angiogenesis in TNBCs, and may represent attractive targets for anti-angiogenic therapy [67]. Increased leptin levels and decreased levels of adiponectin stimulate cell proliferation and angiogenesis and it appears that insulin growth factor-1 (IGF-1) and EGFR modulate the insulin-leptin-adiponectin axis in TNBCs [68]. Unlike angiogenesis, the process of lymphangiogenesis has been less studied in TNBCs. The first and only scientific study that was focused on lymphangiogenesis in TNBCs was carried by Liu et al in 2009 [69]. The node-negative TNBCs specimens used in the study were correlated with a higher intratumor and peritumor lymphatic vascular density (LVD) compared to non-TNBCs specimens, and with a positive lymphatic invasion [69]. Moreover, a positive VEGF-C and VEGF-D expression was noticed, suggesting their implications in the formation of lymphatic vessels in TNBCs [69]. With the exception of the above mentioned research study, VEGF-D is mentioned by Tolaney et al. regarding targeted therapy in metastatic TNBCs, but with no reference to its implications in lymphangiogenesis [70]. The role of other growth factors such as PDGFs/PDGFRs axis and their interactions in promoting angiogenesis and lymphangiogenesis in TNBCs remains to be elucidated.

TNBCs are known to metastasize *via* hematogenous routes [71] and this may be in contradiction with the study of Liu [69] previously mentioned, a study which clearly stated that TNBCs have an active lymphangiogenic process which, normally may favour lymphovascular but not hematogenous dissemination. Currently, the molecular features that differentiate or are able to differentiate lymph node positive TNBCs from lymph node negative TNBCs still remain at a hypothetical level and none of them proved to be useful in the clinical and therapeutic approach of TNBCs patients. But most of the TNBCs cancers have preferentially hematogenous metastases. Besides the high mitotic rate and increased nuclear grade, TNBCs also include pushing border of invasion, frequent tumor necrosis and a large central acellular zone [71]. TNBCs usually exhibit a solid/sheet-like growth pattern and may be associated with an increased lymphocytes infiltrate [71]. Despite the fact that these tumors do not usually metastasize through the lymphatic pathways, TNBCs may be characterized by lymphatic invasion and by an increased LVD [69]. However, not all TNBCs are associated with a poor long term survival, although in a low percentage [71]. EGFR, Src kinase pathway and Cdc42-interacting protein 4 (CIP4) are known to promote TNBCs metastasis [72]. CIP4 inhibition seems to decrease the rate of lung metastasis [72].

Whether metastases posses a similar phenotype as the primary tumor remains an enigma. MicroRNA analysis showed the existence of four TNBCs subclasses with different expression signatures, and it is stated that miRNA signatures contribute to the phenotypic differences of TNBCs and their metastases [73]. A study conducted by Fulga et al. on invasive ductal carcinomas of the breast and their corresponding lymph node metastases, showed that some metastases exhibited the tendency to undergo molecular profile shifting from one subtype to another [74]. Although triple-negative tumor samples were included, the study was mainly focused on luminal A and B which were most frequently detected through immunohistochemical analysis [74]. Do TNBCs metastases maintain a negative profile for all three markers or could they gain positivity for ER, PR or HER2? If this hypothesis was correct, could the molecular shift be drug-dependent or could it simply be hazardous depending on the TNBCs molecular subtype and on the clinical and histopathological status of the patient? In our opinion, all these aspects should be taken into consideration when attempting to test or apply any targeted treatment in TNBCs cases. The molecular TNBCs types, metastases phenotypes, possible molecular profile switches, angiogenesis, lymphangiogenesis and vasculogenic mimicry should constitute reference parameters for TNBCs chemotherapy.

Overall, it should be take in account that TNBC does not apply as a final diagnosis, that it is a conventional designation derived from molecular analyses which does not always reflect a unique clinical presentation and, most of all, a unique histopathological entity. Consequently, TNBC should be stratified accordingly to histopathology, thus excluding some tumor types (adenoid cistyc carcinoma, lobular carcinoma, some subtypes of metaplastic carcinoma) from the subset of TNBC in its conventionally used molecular designation.

## TARGETED THERAPY IN TNBCS

**Figure 1:**
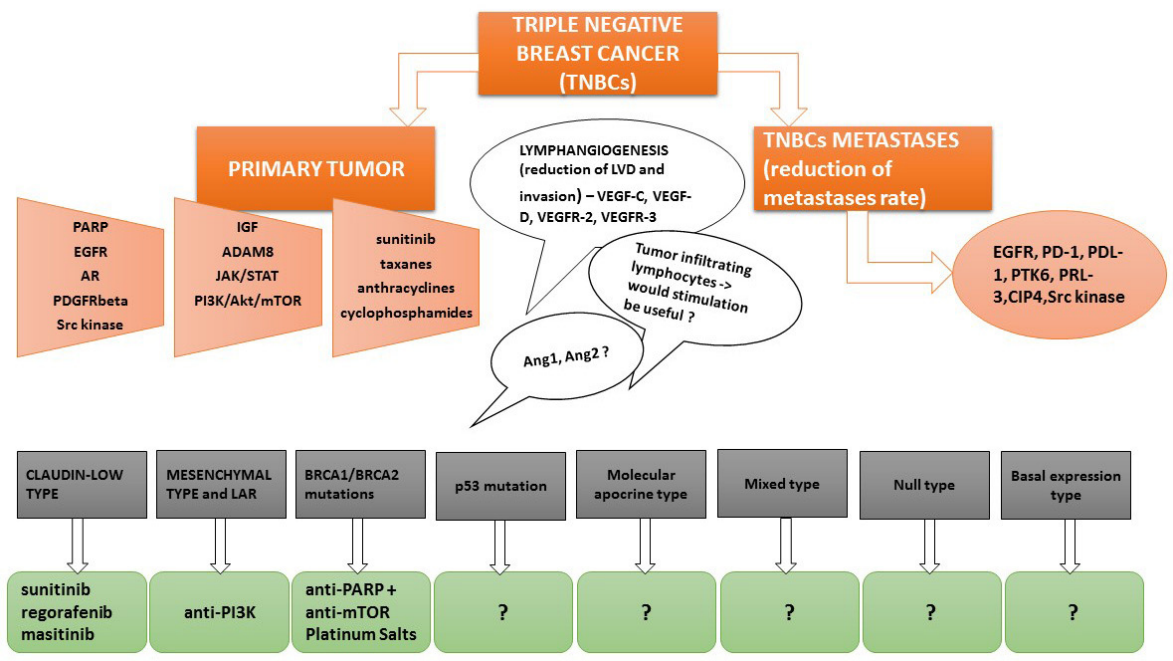
Different potential therapeutic options in TNBCs related to the most neglected but important therapeutic targets

Different potential therapeutic options in TNBCs are summarized in Figure 1. Unlike the luminal types and the HER2 overexpressing type of breast carcinomas, TNBC lacks a specific targeted therapy. However, considering TNBCs heterogeneity, it is possible that BRCA1/2 mutations, along with AR may represent potential molecular targets in TNBCs treatment [75]. Also, Pim-2, a serin/threonine kinase strongly involved in breast cancer metastasis, may become a therapeutic target in TNBCs [76]. HJ-PI01, a Pim-2 inhibitor, seems to induce autophagic cell death and apoptosis thus decreasing malignant proliferation in TNBC cell lines [76]. Shindikar et al. highlighted the anticancer properties of curcumin and resveratrol in TNBCs treatment, however, difficulties regarding their *in vivo* availability, distribution and kinetics along with a poor solubility, limits their routine use in patients [77]. Chemotherapeutic agents such as nab-paclitaxel seems to be beneficial in the treatment of aggressive forms of breast cancer, such as TNBCs and HER2^+^, as well as in elderly and taxane-pretreated women [78].

Tumor heterogeneity causes difficulties in the establishment of a unanimously accepted therapy. Targeted chemotherapy in TNBCs appears to be subclass-dependant. It is well known that some TNBCs types are associated with a much worse prognosis, such as the claudin-low type [79]. Molecular targets such as VEGF, EGFR, DNA repair pathway, androgen and NOTCH pathways have been proposed throughout the years [80, 81]. VEGF-A is not a promising molecular target in TNBCs. Schneider et al. have shown that VEGF-A amplification in TNBCs was associated with a poor outcome and that bevacizumab based treatment in TNBCs lead to a poor overall survival [66]. Anti-VEGF-C and anti-VEGF-D treatment could significantly reduce LVD and lymphovascular invasion considering the important implications of these receptors in breast cancer lymphangiogenesis [69]. Through their associated receptors, VEGFR-2 and VEGFR-3, VEGF-C and -D are one of the most potent promoters of lymphangiogenesis in a wide range of cancers. Moreover, the inactivation of the BRCA pathway along with immunotherapy seem to be pertinent trials for clinical practice in the future, considering the fact that most TNBCs are associated with BRCA mutations and the presence of immune infiltrates in TNBCs possesses both a predictive and a prognostic role [82]. The combination of PARP inhibitors with mTOR inhibitors appear to be an effective therapeutic strategy in TNBCs patients presenting BRCA mutations [43, 82, 83]. For the treatment of metastatic TNBCs, checkpoint inhibitors (PD-1 and PD-L1 inhibitors) are already emerging as promising therapeutic agents [82, 84]. However, the role PD-L1, the ligand of PD-1, in TNBCs seems quite controversial, as other papers state that its expression is frequently seen in TNBCs and that it is associated with a better disease-free survival [85].

Although bevacizumab-based anti-angiogenic therapy has highly improved the oncologic treatment in several types of cancers, it seems to be ineffective in TNBCs. No positive responses were documented in patients that presented VEGF-A amplification following bevacizumab-based treatment, the progression-free survival being rated as inferior according to Schneider et al. [66]. PDGFRbeta inhibitors however seem to prove themselves beneficial in TNBCs treatment [62, 86]. Therapeutic agents such as Sunitinib, Regorafenib and Masitinib have shown their utility in inhibiting cell migration, proliferation and metastasis in the claudin-low subtype [86].

If TNBCs treatment depends on the molecular profile of the tumor, the same statement may be postulated for TNBCs metastasis. In a recently published article, Kulka et al. stated that ‘characterization of the metastases is necessary for appropriate treatment and planning’, with reference to central nervous system metastases [87]. Currently, it seems that TNBCs metastases treatment are studied parallel with different biomarkers of identification. Due to the incomplete elucidation of the molecular profile of TNBCs metastases, a standardized therapy is not yet available in clinical practice. However, protein tyrosine kinase 6 (PTK6), a protein tyrosine kinase expressed in approximately 70% of TNBCs, may constitute a potential target for metastases suppression [88]. Also, the metastatic potential of TNBCs may be successfully reduced through metastasis-associated phosphatase of regenerating liver-3 (PRL-3) blocking, a metastasis-promoting phosphatase [89]. Mathe et al. have identified no more no less than 83 genes that positively correlate with lymph node metastasis in TNBCs and may be used as molecular targets or prognostic indicators [90].

Despite the numerous pharmacological trials carried in the field of triple-negative breast cancers, trials that are not yet clinically beneficial, one thing is certain: TNBCs continue to arouse the interest of both researchers and physicians worldwide. It may be stated that the majority of papers focused on triple-negative breast cancers, their metastases and targeted treatment have been published between the years 2015-2016. In addition, most scientific studies regarding TNBCs chemotherapy have been published in 2016 or are ahead of print as we write. However, much more research is required in order to elucidate the complex molecular insights of primary TNBCs and of their metastases, whether lymph node or distant metastases. Also, the clarification of TNBCs carcinogenesis is an essential issue for the establishment of a proper treatment strategy. Angiogenesis and lymphangiogenesis in TNBCs require further analysis, considering the fact that both processes imply the participation of a great range of growth factors and signaling molecules. These issues have an important impact in TNBCs treatment as it is known that the use of a combination of several chemotherapeutic drugs may be associated with a high toxicity. Therefore, the question: ‘Could TNBC be subject to a standardized, unanimously accepted therapeutic strategy or is it strictly subclass-dependent?’ remains to be further investigated.
